# Remarkable Response to Immunotherapy and VEGFR Inhibitor in Xp11 Translocation Renal Cell Carcinoma in a 17‐Year‐Old Adolescent

**DOI:** 10.1155/carm/7267084

**Published:** 2026-07-21

**Authors:** Piyarat Limpawittayakul, Narongchai Teerayathanakul

**Affiliations:** ^1^ Department of Medical Oncology, Chulabhorn Hospital, Chulabhorn Royal Academy, Bangkok, Thailand, cra.ac.th; ^2^ Department of Pathology, Chulabhorn Hospital, Chulabhorn Royal Academy, Bangkok, Thailand, cra.ac.th

**Keywords:** case report, immunotherapy, renal cell carcinoma, Xp11 translocation

## Abstract

**Background:**

Xp11 translocation renal cell carcinoma (tRCC) is a rare and aggressive renal cancer characterized by gene fusion involving *TFE3*. It accounts for < 5% of renal cancers and shows variable responses to conventional antivascular endothelial growth factor therapies. Optimal treatment strategies for metastatic cases have not been determined.

**Objective:**

To report the clinical presentation, molecular diagnosis, and therapeutic response of an adolescent patient with metastatic Xp11 tRCC treated with pembrolizumab and lenvatinib.

**Methods:**

A 17‐year‐old female presented with a large left renal mass, gross hematuria, extensive metastases to the supraclavicular, mediastinal, and intra‐abdominal lymph nodes, and pleural nodules. Immunohistochemistry confirmed transcription factor E3 (TFE3) positivity, and next‐generation sequencing identified an *ASPSCR1–TFE3* fusion. Following renal embolization for local control, the patient received systemic therapy (pembrolizumab at 200 mg/dose given every 3 weeks and lenvatinib at 10 mg–20 mg/day).

**Results:**

The patient showed rapid clinical improvement, with her Eastern Cooperative Oncology Group Performance Status Scale grade improving from 3 to 1 after the initial cycles. CT imaging after the sixth cycle demonstrated a marked reduction in the size of the primary tumor and in the sizes and numbers of metastatic lesions, involved lymph nodes, and pleural nodules. The patient recovered to normal well‐being.

**Conclusion:**

Pembrolizumab combined with lenvatinib showed significant antitumor activity in an adolescent with advanced *ASPSCR1*–*TFE3* fusion Xp11 tRCC. These findings support the utility of molecular profiling of renal tumors in young adults and suggest that this combination is an effective treatment option for this subtype of renal cancer.

## 1. Introduction

Renal cell carcinoma (RCC) encompasses a variety of malignancies with different histological subtypes. It is uncommon in children and adolescents, accounting for < 5% of all pediatric renal tumors. Xp11 translocation RCC (Xp11 tRCC) is a distinct molecular and pathological RCC subtype characterized by translocations involving *TFE3* at the Xp11.2 chromosomal locus that result in gene fusions—most commonly *ASPSCR1*‐*TFE3* or *PRCC*‐*TFE3*—which lead to the overexpression of TFE3 [1–4]. In addition, genes associated with mitochondrial respiration are upregulated in tRCC [5]. Xp11 tRCC was first recognized as a distinct subtype in the World Health Organization’s 2004 classification of kidney tumors [6]. In 2022, it was reclassified as a molecularly defined RCC: TFE3‐rearranged RCC [7].

Xp11 tRCC primarily affects children and adolescents; however, it is increasingly being reported in young adults, in whom it often exhibits more aggressive clinical behavior, with higher rates of metastasis at presentation and poorer outcomes compared to pediatric cases [3]. It accounts for about 30% of pediatric RCCs and 1%–5% of adult RCCs. This type of tumor metastasizes to bone, the lungs, the liver, and distal lymph nodes [4].

Due to the rarity of Xp11 tRCC and limited prospective data, optimal treatment strategies for managing this subtype of kidney cancer have not been determined. In metastatic cases, variable and limited responses to conventional antivascular endothelial growth factor (VEGF) therapies are often observed [8].

Here, we present a case of advanced Xp11 tRCC in a 17‐year‐old patient who subsequently experienced a remarkable and durable response to immune checkpoint inhibitor (ICI)‐based immunotherapy. This case highlights the importance of performing a molecular diagnosis when young patients present with RCC and the potential role that immunotherapy may play in the treatment of this aggressive RCC subtype.

## 2. Case Presentation

In November 2024, a 17‐year‐old Cambodian female with no known comorbidities presented with upper left quadrant abdominal pain. The pain was dull, radiated to her back, and made it difficult for her to lie down and worsen over time. Two to 3 months prior, the patient experienced anorexia and lost 10 kg in 2 months. She developed gross hematuria and a palpable neck mass. The patient was determined to be grade 3 on the Eastern Cooperative Oncology Group (ECOG) Performance Status Scale. She had no family history of malignancy.

A contrast‐enhanced CT scan revealed a 12.4 × 10.3 × 14.3 cm heterogeneous enhancing mass involving the upper to middle section of the left kidney (Figure 1). The lesion extended beyond the perinephric space, causing pressure effects on the spleen, stomach, and pancreas. Additionally, there were enhancing lymph nodes in the gastrohepatic, hepatoduodenal, para‐aortic, aortocaval, and left common iliac regions that were up to 7.1 × 5.2 cm in size; intrathoracic nodes that were up to 3.9 × 1.6 cm in size; and left supraclavicular nodes that were up to 5.2 × 3.1 cm in size (Figure 2). As shown in Figure 3, multiple pleural nodules/masses that were up to 5.3 × 4.6 cm in size and a moderate amount of pleural effusion were also observed. A bone scan showed no evidence of bone metastasis.

**FIGURE 1:**
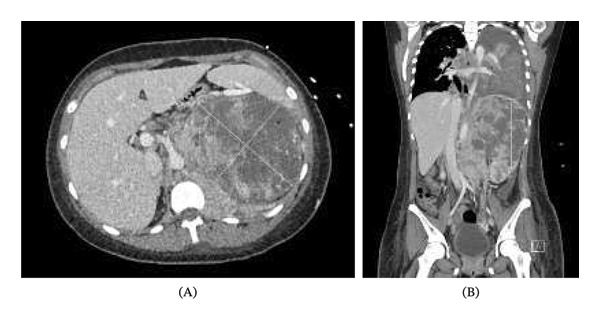
CT scanning revealed a large, heterogeneous, enhancing mass in the midsection of the left kidney that extended beyond the perinephric space. (A) Cross‐sectional view. (B) Coronal view.

**FIGURE 2:**
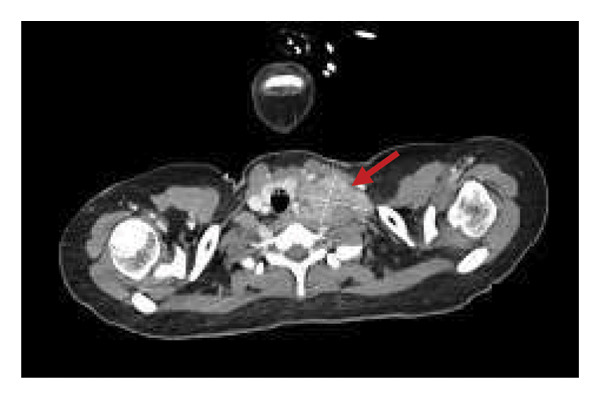
CT image showing left supraclavicular lymphadenopathy.

**FIGURE 3:**
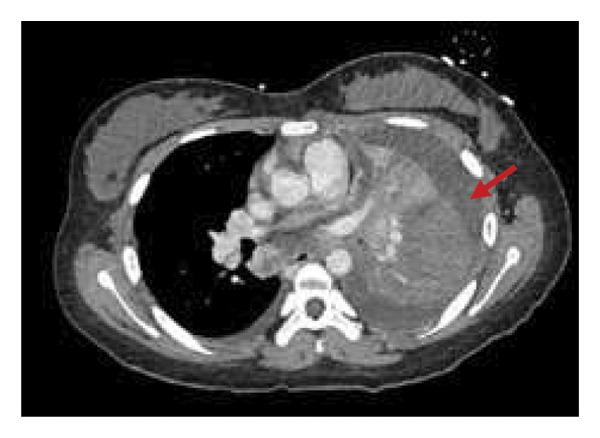
CT image showing multiple pleural nodules/masses and moderate pleural effusion.

When the left supraclavicular lymph nodes were biopsied and analyzed, a large epithelial neoplasm with solid nest rearrangement was identified. Prominent nucleoli with chromatin rimming were present, as well as abundant eosinophilic cytoplasm with fine granular features. The stromal component in the interstitium was scarce (Figures 4A,B). These features are not typically observed in clear cell or chromophobe RCC.

**FIGURE 4:**
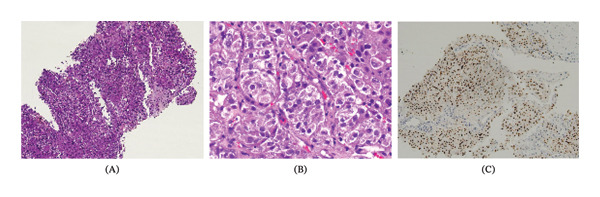
(A) Left supraclavicular lymph node sample stained with hematoxylin and eosin (H&E, 4X). (B) Prominent nucleoli with chromatin rimming and abundant eosinophilic cytoplasm with fine granular features are evident (H&E, 20X). (C) Transcription factor E3 (TFE3) was strongly expressed in the nuclei of the tumor cells.

Tumor cells subjected to immunohistochemistry (IHC) showed CD10, PAX8, P504, and TFE3 expression; however, the following molecules were not detected: AE1/AE3, CK7, CK20, TTF‐1, CDX‐2, CAM5.2, HepPar1, CD117, vimentin, inhibin, CD99, melan A, and epithelial membrane antigen (EMA) (Figure 4C). Next‐generation sequencing of tumor cells revealed a translocation of the Xp11 gene with *ASPSCR1* located on chromosome 17 (Figure 5). No additional genetic variants were detected.

**FIGURE 5:**
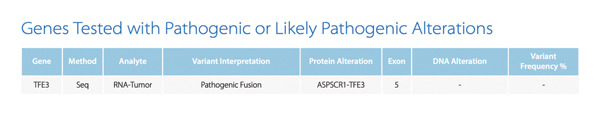
Next‐generation sequencing of tumor cells revealed a translocation of the Xp11 gene with *ASPSCR1* located on chromosome 17.

The patient had iron deficiency anemia due to chronic blood loss via hematuria. Thus, the treatment regimen included a blood transfusion and ferrous fumarate supplementation. Excess pleural fluid was drained, which resulted in symptom relief. The fluid was determined to be an exudate and pleocytosis, with monocytes being the predominant cell type, and atypical cells were observed in the cell block.

After discussing the case at a tumor board meeting, local control options were considered to manage the primary tumor due to chronic gross hematuria and left flank pain. However, surgical intervention was not feasible due to the advanced stage of the tumor. Because no other methods could stop the severe bleeding, a renal angiogram and embolization were performed, which successfully improved the gross hematuria. However, the patient’s pain persisted, necessitating the use of morphine for pain relief.

Pembrolizumab (a PD‐1 inhibitor) and lenvatinib (a tyrosine kinase inhibitor [TKI]) were administered. Pembrolizumab was initially administered at 200 mg/dose given every 3 weeks, while lenvatinib was initially administered at a lower dose of 10 mg/day due to the patient’s ECOG Performance Status Scale grade of 3. The systemic treatment was very well tolerated. Three weeks after the first treatment cycle, significant improvements were observed, including smaller cervical lymph nodes, improved dyspnea, pain relief, and increased appetite. For the second cycle, pembrolizumab was administered at the same dose, while lenvatinib was increased to 14 mg/day. The patient’s condition further improved after the second cycle (achieving ECOG Performance Status Scale Grade 1). Subsequently, lenvatinib was administered at a maximal dose of 20 mg in the third cycle. The patient exhibited good tolerance of this dose. To date (June 2026), 22 cycles of pembrolizumab and lenvatinib (20 mg/day) have been completed, and we plan to continue this treatment until disease progression or up to 35 cycles. The patient experienced an adverse effect, specifically drug‐induced hypothyroidism which was detected during the ninth cycle. However, levothyroxine supplementation managed this condition without interrupting the systemic treatment.

A CT scan performed after the sixth and twelfth cycles showed a marked reduction in the size and number of multiple left supraclavicular, mediastinal, and intra‐abdominal lymph nodes. Reductions in pleural nodules and thickening were also evident. The left kidney tumor mass decreased in size; however, this may have been due to the effects of embolization (Table 1). The patient recovered well and returned to baseline condition.

**TABLE 1:** Remarkable treatment response of measurable lesions in this patient.

	Baseline CT scan	Post‐6th cycle CT	Post‐12th cycle CT
Left supraclavicular lymph node	5.2 × 3.1 cm	4.3 × 2.6 cm	2.0 × 2.3 cm
Pleural nodules	5.3 × 4.6 cm, marked pleural effusion	1.2 × 1.0 cm, minimal pleural effusion	1.2 × 1.0 cm, no pleural effusion
Left kidney mass	12.4 × 10.3 × 14.3 cm	7.1 × 6.8 × 8.9 cm	6.9 × 6.6 × 8.4 cm
Intra‐abdominal lymph nodes	7.1 × 5.2 cm	4.7 × 5.1 cm	4.2 × 4.9 cm

## 3. Discussion

In Xp11 tRCC, *TFE3* on chromosome *X* fuses with other genes, including *ASPSCR1*, *SFPQ*, *NONO*, *PRCC*, *RBM10*, and *MED15*, resulting in upregulation of TFE3 [9]. The most common fusions are *ASPSCR1*–*TFE3*, which is associated with t(X; 17) (p11; q25) translocation, and *PRCC–TFE3*, which is associated with t(X; 1) (p11; q21) translocation [2]. Tumors with *ASPSCR1*–*TFE3* fusions develop earlier (median age: 21 years), are more likely to have progressed to an advanced stage at diagnosis (i.e., involve regional lymph nodes [N1 disease] and distant metastases [M1 disease]), and have worse outcomes than those with other gene fusions [10].

The classic morphological hallmark of Xp11 tRCC is the presence of tumor cells with abundant eosinophilic or clear cytoplasm arranged in papillary or micropapillary structures, often accompanied by psammoma bodies. Typically, cells with an *ASPSCR–TFE3* fusion have a higher clear‐to‐eosinophilic cytoplasm ratio than cells with other fusions and exhibit discontinuous cell boundaries, vesicular nuclei, and obvious nucleoli [11]. However, only about half of the TFE3‐positive RCCs display this classic morphology. Furthermore, even tumors with typical features may be misdiagnosed as other RCC subtypes, such as clear cell or papillary RCC, particularly in adults. Therefore, it is essential to confirm the presence of TFE3 via IHC and to perform molecular analysis to ensure an accurate diagnosis of Xp11 tRCC [12]. Xp11 tRCC cells typically also express PAX2, PAX8, CD10, and cathepsin K, while markers such as vimentin, cytokeratin, EMA, and CK7 are usually underexpressed in Xp11 tRCC cells [13].

Argani et al. [14] showed that detecting TFE3 via IHC was a highly sensitive (97.5%) and specific (99.6%) method for diagnosing Xp11 tRCC. However, others have reported broader ranges, with sensitivity values of 70%–85% and specificity values of 57%–95%; this is likely due to technical variability [15]. Moderate‐to‐strong and diffuse nuclear immunoreactivity to the C‐terminal portion of TFE3 has also been shown to be diagnostically meaningful, although weak staining may be overlooked, particularly in tumors with classic histological features [16]. As such, genetic confirmation via TFE3 break‐apart fluorescence in situ hybridization (FISH) remains the gold standard for diagnosing Xp11 tRCC. In cases involving inverted *X* chromosome translocations, FISH may yield equivocal results, making TFE3 detection via IHC a valuable complementary method. Therefore, the combined use of IHC and FISH is the preferred approach for achieving diagnostic accuracy [4].

Given the rarity of tRCC and a limited grasp of its pathogenesis, standardized treatment protocols remain undefined. Yan et al. [17] explored the efficacy of various therapies, including anti‐VEGFR agents, mTOR inhibitors, and immunotherapy. Their findings indicated varying objective response rates (ORR) among treatments: axitinib led at 33.3%, followed by pazopanib (20.0%), sunitinib (7.1%), and sorafenib (6.7%), while one patient responded partially to cabozantinib. For the entire cohort, the median progression‐free survival (PFS) was 7.4 months with an overall survival (OS) of 17.9 months. Notably, two patients receiving first‐line combination therapy of VEGFR‐TKI and anti‐PD‐1 inhibitors achieved extended PFS durations of over 16.6 and 25.6 months [18].

Immunotherapy has been shown to provide clinical benefits in cases of clear cell RCC, and its efficacy is now being actively investigated in nonclear cell RCC. For example, in the single‐arm phase 2 KEYNOTE‐B61 trial [17], the efficacy of pembrolizumab combined with lenvatinib was evaluated as a first‐line therapy for advanced nonclear cell RCC. The trial included six patients (4%) with tRCC. The overall ORR was 49%, and a complete response was achieved in 6% of the participants. In the subgroup of patients with tRCC, the ORR was 67%. The median PFS was 18 months, and the median OS was not reached. Another single‐arm phase 2 study [19] evaluated the combination of nivolumab and cabozantinib as a first‐ or second‐line therapy for the treatment of advanced nonclear cell RCC. Among the 40 participants, there were two patients (5%) with TFE3 translocation‐associated RCC. In the first‐line setting, the ORR for the entire cohort was 54% and 50% for the tRCC subgroup. The median PFS and OS in the first‐line setting were 11 months and 28 months, respectively.

The therapeutic outcomes of combining immunotherapy with VEGFR inhibitors appear to be significantly influenced by specific molecular signatures. In particular, the clinical benefits of ICI‐based regimens are most evident in patients harboring the ASPSCR1–TFE3 fusion subtype. This genetic variant is defined by an up‐regulation in pathways of angiogenesis, stromal remodeling, and cell proliferation. Despite their highly angiogenic profile, ASPSCR1–TFE3 tumors frequently demonstrate resistance to VEGFR‐TKI monotherapy. This suboptimal response is likely linked to the dense extracellular matrix (ECM) characteristic of these tumors, which may function as a barrier to therapy. Furthermore, the ECM and collagen play pivotal roles as immunomodulators within the tumor microenvironment (TME), modulating immune checkpoint expression and consequently determining the overall efficacy of ICI therapy [10].

## 4. Conclusion

In this case study, a 17‐year‐old female with Xp11 tRCC exhibited a remarkable response to immunotherapy. Our findings highlight the potential of immunotherapy in combination with VEGFR‐TKI therapy as a treatment option for this rare form of RCC, similar subtypes of RCC, and other forms of cancer.

## Author Contributions

Conceptualization, Piyarat Limpawittayakul; pathologist, Narongchai Teerayathanakul.

## Funding

We are pleased to acknowledge the support extended by the Chulabhorn Royal Academy, Thailand, which provided funding for the completion of this study.

## Disclosure

All authors have read and agreed to the published version of the manuscript.

## Ethics Statement

This study was meticulously conducted with strict adherence to the ethical principles outlined in the Declaration of Helsinki guidelines governing research involving human subjects. The study protocol underwent thorough review and received approval from the Ethics Committee for Human Research at the Chulabhorn Research Institute (Certificate No.043/2568). Informed consent was provided by the patient.

## Consent

Consent for publication was provided by the patient.

## Conflicts of Interest

The authors declare no conflicts of interest.

## Data Availability

The data that support the findings of this study are available on request from the corresponding author. The data are not publicly available due to privacy or ethical restrictions.
